# The role of novel poly (ADP-ribose) inhibitors in the treatment of locally advanced and metastatic Her-2/neu negative breast cancer with inherited germline BRCA1/2 mutations. A review of the literature

**DOI:** 10.25122/jml-2020-0132

**Published:** 2021

**Authors:** Lucian Pop, Ioan Suciu, Olivia Ionescu, Nicolae Bacalbasa, Paris Ionescu

**Affiliations:** 1. Department of Obstetrics and Gynecology, Alessandrescu-Rusescu National Institute of Mother and Child Health, Bucharest, Romania; 2. Department of General Surgery, Floreasca Emergency Hospital, Bucharest, Romania; 3. Department of Obstetrics and Gynecology, Carol Davila University of Medicine and Pharmacy, Bucharest, Romania; 4. Department of Obstetrics and Gynecology, South Nurnberg Hospital, Nurnberg, Germany; 5. Department of General Surgery, Carol Davila University of Medicine and Pharmacy, Bucharest, Romania; 6. Department of Obstetrics and Gynecology, Ovidius University, Constanta, Romania

**Keywords:** breast, cancer, PARP inhibitors, BC – Breast Cancer, DSB – Double-Strand Break, HER-2/neu – Overexpression of the Herceptin Receptor, HR – Homologous Recombination, OS – Overall Survival, PARP – Poly (adenosine diphosphate-ribose) Polymerase, PARPi – Poly (adenosine diphosphate-ribose) Polymerase Inhibitors, TNBC – Triple-Negative Breast Cancer, PFS – Progression-Free Survival

## Abstract

The use of the PARP inhibitors (PARPi) in the treatment of breast cancer (BC) with germine mutations has evolved over the years, and further research has been done in order to broaden the horizon of this treatment strategy. Therefore the aim of this paper is to review the efficiency of PARPi in the treatment of BRCA 1/2-mutated locally advanced and metastatic Her-2/net negative BC mentioning their side effects, mechanism of resistance and future directions. Inhibition of PARP transforms single-strand breaks into double-strand breaks (DBS), the accumulation of the latter causing cell death (cell apoptosis). The Olympia AD phase III trial demonstrated a statistically significant progression-free survival rate (PFS) when using the PARPi olaparib in metastatic BC with germline BRCA1/2 mutations without any benefit of the overall survival rate. PARPi therapy is associated with acceptable responsive rates and progression-free survival rates in locally advanced and metastatic BRCA1/2 associated BC through mechanisms that enhance and increase the sensitivity to chemotherapeutic or target agents as they induce a synthetic lethality and cell apoptosis. The side effects are not significant, the most adverse effects being related to the hematological and gastrointestinal systems. Olaparib is currently approved in the first-line treatment of BRCA1/2 mutated Her-2/neu negative metastatic BC at an oral dose of 300 mg twice daily, while Talazoparib represents a category one recommendation in locally advanced and metastatic Her-2/neu negative BC in women with central nervous system metastases.

## Introduction

Five to 10% of breast cancers (BC) are hereditary due to germline mutations in the two of the most studied genes - BRCA 1 and BRCA 2, which are known to be located on chromosomes 17 and 13, respectively [1, 2]. Mutations in these two genes have an autosomal dominant inheritance pattern [2] and have been proved to be associated with an estimated predisposition to develop BC of almost 84% [2]. BRCA 1 and BRCA 2 play a tumor suppression role and are involved in the homologous recombination (HR) repair mechanism of damaged double-stranded DNA of the malignant cells [3]. The HR allows the double-stranded DNA to reestablish its initial healthy genetic sequence using repair pathways that are not causing continuous errors in the DNA sequence [4]. In cells with mutations of the BRCA 1 and BRCA 1 genes, the HR mechanism is either absent or impaired, leading to defective genetic sequences and activation of oncogenes that, in turn, stimulate the proliferation of defective cells with genetically mutated DNA sequences [5]. 

As a result of damaging the DNA cell structure, the PARP enzymes are activated, resulting in an enhancement of the process of ADP ribosylation and accumulation of single-stranded repaired DNA, which are ready to replicate and thus determine the survival of the cells [6, 7]. The therapies targeting the inhibition of PARP aim to hinder the process of ADP ribosylation, hence preventing the repair mechanism promoted by the PARP enzymes [8]. The process of replication is interrupted, causing replication splits [9], which disintegrates and forms a double-strand break (DSB). The DSBs can be repaired by the process of HR, but, as mentioned before, in BRCA 1/2 mutated cells, HR is deficient. Therefore, the accumulation of DBS will cause the death of the cells (apoptosis) [10]. 

BRCA1/2 mutated cells have been demonstrated to lack the HR mechanism, and this means the DBS cannot be repaired correctly, thus making these cells a target to the therapy with PARP inhibitors (PARPi) [3, 11]. 

Furthermore, the confirmation that some triple-negative breast cancers (TNBC) are phenotypically similar to breast tumors with BRCA1 mutations has aroused the interest of researchers to study the effectiveness of PARP inhibitors in both BC with BRCA1/2 germline mutations and TNBC [12]. 

This paper aims to present the effectiveness of PARPi by presenting the recent developments and clinical trials that involved these target agents along with their possible combination with other cytotoxic or target agents, as well as their side effects and mechanisms of resistance.

## Clinical trials of PARPi in BRCA-associated breast cancers

The first study on the efficiency of PARPi in breast cancer was conducted by Bryant *et al.* [13] and Farmer *et al.* [14] and included women with breast tumors that are not necessarily BRCA1/2 mutated but resemble BRCA-mutated BC such as TNBC phenotypically. The results were discouraging. Therefore, the group of patients has been extended to germline BRCA 1/2 mutated BC. Fong *et al.* [15] showed in their phase 1 clinical trial that the therapy with olaparib was associated with a decrease in the level of tumor markers as well as a progression-free interval of at least four months in 63% of women while the single-use of talazoparib proved to have a clinical benefit in 33% of women with BRCA 1/2 mutated BC [16]. A response rate ranging from 33% to 41% after olaparib 400 mg orally as monotherapy with acceptable side effects has been shown in the phase 2 trial conducted by Tutt *et al.* [10] and Audeh *et al.* [17] in both TNBC and negative BC. On the contrary, a lower response rate of only 12.9% after using olaparib as monotherapy has been observed in the past two trials of Kaufmann *et al.* [9], a chemotherapy regimen used before olaparib, being supposed to be responsible for the resistance to olaparib in the subgroup of women who initially received chemotherapy before olaparib. 

A phase III international, multicenter, randomized trial - OlympiaAD- tested the efficacy of PARPi in the metastatic setting of BRCA1/2-associated BC. The 302 women have randomly received either olaparib 300 mg two tablets daily or monotherapy with eribulin, capecitabine or vinorelbine [18]. The olaparib group registered an objective response of 59.9% compared to 28.8% in the chemotherapy group, while the overall survival rates were similar between the two groups by approximately 19 months [18]. A dose reduction of olaparib, primarily due to anemia and secondly due to nausea, neutropenia, fatigue, and diarrhea, was necessary in 14% of cases. In comparison, interruption or discontinuation of treatment was observed in 33% of cases [4, 18].

PARPi also showed encouraging results when combined with other cytotoxic drugs as they possess a synergic effect as well as enhancing the effect of a chemotherapy regimen [2]. Veliparib increases the cytotoxic effect of temozolomide, this combination being associated with a complete response in 50% of women with germline BRCA-associated BC as well as a response rate of 22% [19]. Response rates up to 73%, including stable disease, partial and complete responses, have been recorded in regiments combining olaparib with cisplatin and carboplatin and topotecan [20-22]. Significant hematologic toxicity with grade 3 neutropenia has been reported when combining olaparib with paclitaxel [23].

## PARPi in the clinical setting

Currently, PARPi are approved in the treatment of metastatic Her-2/neu negative BC with BRCA1/2 mutations with previous chemotherapy treatment. For women with advanced hormone receptor-positive BC, initial endocrine therapy should be completed before starting the therapy with PARPi [24]. The recommended dose is 300 mg orally, twice daily with or without food [4, 18, 24]. The primary metabolization is through the hepatic pathway, while the metabolites are secreted through urine or feces [24]. The mean half-life is approximately 14.9 hours, and the combination with CYP3A4 inducers should be avoided [18, 24].

The main side effects of olaparib therapy refer to the myelodysplastic syndrome and acute myeloid leukemia, which were observed in 1.5% of women who also previously received chemotherapy with platinum-based agents [4]. Another rare side effect is pneumonitis, reported in less than 1% of women [18]. The routine monitoring includes a complete blood count, renal and hepatic function tests, as well as a pregnancy test [4] as conception is not allowed during the treatment with PARPi or in the next 6 months after treatment completion [4].

The development of resistance to PARPi is a major field of interest in the specialized literature [3, 25]. The two primary mechanisms of developing resistance to PARPi involve new mutations in the mutated BRCA1/2 genes due to the genomic instability caused by PARPi and a possible reduction of PARPi intracellular concentrations as a result of increased drug efflux [25-27]. Cross-resistance between olaparib and platinum-based agents has been reported by Audeh *et al.* [17] and Kaufmann *et al.* [9] with better response rates to olaparib in women without previous platinum-based chemotherapy. On the other side, the development of resistance to PARPi seems to interfere with the sensitivity to platinum agents as the latter persists after PARPi therapy has been interrupted due to the development of resistance [28]. One method to defeat resistance to PARPi involves the use of medication that blocks the efflux pumps, a mechanism that might increase the cell susceptibility to PARP inhibition [29]. Further research is needed in order to identify the subset of patients who would benefit most from PARPi in combination with chemotherapy regimens as the majority of the combination drugs share a similar mechanism of action and resistance pathway to PARPi [30]. 

## Future directions and conclusions 

Ongoing clinical trials are trying to identify a possible benefit from PARPi in breast tumors with somatic BRCA1/2 mutations combined with radiotherapy in the adjuvant setting(31). Other future directions include the combination of PARPi with immunological agents such as atezolizumab, durvalumab, and tremelimumab in HER2-negative metastatic breast cancer [32]. The use of talazoparib has been shown to be associated with a longer median PFS compared to other agents such as capecitabine, eribulin, gemcitabine, or vinorelbine. Anemia was the only significant side effect that was reported [33]. Talazoparib is currently recommended as a first-line option in patients with advanced or metastatic Her-2/neu negative BC with associated germline BRCA1/2 mutations [34]. Olaparib showed clinical benefits in metastatic TNBC with lesser side effects than capecitabine. However, additional research is needed to identify other somatic BRCA1/2 mutations that might respond to treatment with PARPi in combination with chemotherapy and/or anti-angiogenic agents or immune inhibitors. The identification of valid biomarkers that predict the response to PARPi and methods to overcome resistance to PARP inhibitors will improve the therapeutic spectrum of these agents, introducing them as an efficient therapeutic option in locally advanced and metastatic BC with germline BRCA1/2 mutations.

## Acknowledgments

### Conflict of interest

The authors declare that there is no conflict of interest.
